# Targeting synovial fibroblast proliferation in rheumatoid arthritis (TRAFIC): an open-label, dose-finding, phase 1b trial

**DOI:** 10.1016/S2665-9913(21)00061-8

**Published:** 2021-03-09

**Authors:** Arthur G Pratt, Stefan Siebert, Michael Cole, Deborah D Stocken, Christina Yap, Stephen Kelly, Muddassir Shaikh, Amy Cranston, Miranda Morton, Jenn Walker, Sheelagh Frame, Wan-Fai Ng, Christopher D Buckley, Iain B McInnes, Andrew Filer, John D Isaacs

**Affiliations:** a Translational and Clinical Research Institute, Newcastle University, Newcastle upon Tyne, UK; b Population Health Sciences Institute, Newcastle University, Newcastle upon Tyne, UK; c Clinical Trials Unit, Newcastle University, Newcastle upon Tyne, UK; d Newcastle upon Tyne Hospitals NHS Foundation Trust, Newcastle upon Tyne, UK; e Institute of Infection, Immunity and Inflammation, University of Glasgow, Glasgow, UK; f Leeds Institute of Clinical Trials Research, University of Leeds, Leeds, UK; g Clinical Trial and Statistics Unit, The Institute of Cancer Research, London, UK; h Department of Rheumatology, Barts Health NHS Trust, London, UK; i Department of Rheumatology, James Cook University Hospital, Middlesbrough, UK; j Cyclacel, Dundee, UK; k National Institute for Health Research Birmingham Biomedical Research Centre, University Hospitals Birmingham NHS Foundation Trust and Institute of Inflammation and Ageing, University of Birmingham, Birmingham, UK

## Abstract

**Background:**

Current rheumatoid arthritis therapies target immune inflammation and are subject to ceiling effects. Seliciclib is an orally available cyclin-dependent kinase inhibitor that suppresses proliferation of synovial fibroblasts—cells not yet targeted in rheumatoid arthritis. Part 1 of this phase 1b/2a trial aimed to establish the maximum tolerated dose of seliciclib in patients with active rheumatoid arthritis despite ongoing treatment with TNF inhibitors, and to evaluate safety and pharmacokinetics.

**Methods:**

Phase 1b of the TRAFIC study was a non-randomised, open-label, dose-finding trial done in rheumatology departments in five UK National Health Service hospitals. Eligible patients (aged ≥18 years) fulfilled the 1987 American College of Rheumatology (ACR) or the 2010 ACR–European League Against Rheumatism classification criteria for rheumatoid arthritis and had moderate to severe disease activity (a Disease Activity Score for 28 joints [DAS28] of ≥3·2) despite stable treatment with anti-TNF therapy for at least 3 months before enrolment. Participants were recruited sequentially to a maximum of seven cohorts of three participants each, designated to receive seliciclib 200 mg, 400 mg, 600 mg, 800 mg, or 1000 mg administered in 200 mg oral capsules. Sequential cohorts received doses determined by a restricted, one-stage Bayesian continual reassessment model, which determined the maximum tolerated dose (the primary outcome) based on a target dose-limiting toxicity rate of 35%. Seliciclib maximum concentration (C_max_) and area under the plasma concentration time curve 0–6 h (AUC_0–6_) were measured. This study is registered with ISRCTN, ISRCTN36667085.

**Findings:**

Between Oct 8, 2015, and Aug 15, 2017, 37 patients were screened and 15 were enrolled to five cohorts and received seliciclib, after which the trial steering committee and the data monitoring committee determined that the maximum tolerated dose could be defined. In addition to a TNF inhibitor, ten (67%) enrolled patients were taking conventional synthetic disease modifying antirheumatic drugs. The maximum tolerated dose of seliciclib was 400 mg, with an estimated dose-limiting toxicity probability of 0·35 (90% posterior probability interval 0·18–0·52). Two serious adverse events occurred (one acute kidney injury in a patient receiving the 600 mg dose and one drug-induced liver injury in a patient receiving the 400 mg dose), both considered to be related to seliciclib and consistent with its known safety profile. 65 non-serious adverse events occurred during the trial, 50 of which were considered to be treatment related. Most treatment-related adverse events were mild; 20 of the treatment-related non-serious adverse events contributed to dose-limiting toxicities. There were no deaths. Average C_max_ and AUC_0–6_ were two-times higher in participants developing dose-limiting toxicities.

**Interpretation:**

The maximum tolerated dose of seliciclib has been defined for rheumatoid arthritis refractory to TNF blockade. No unexpected safety concerns were identified to preclude ongoing clinical evaluation in a formal efficacy trial.

**Funding:**

UK Medical Research Council, Cyclacel, Research into Inflammatory Arthritis Centre (Versus Arthritis), and the National Institute of Health Research Newcastle and Birmingham Biomedical Research Centres and Clinical Research Facilities.

## Introduction

Chronic, painful inflammation, synovial hypertrophy, and associated structural and functional deterioration of peripheral joints are hallmarks of rheumatoid arthritis. They account for the considerable burden of disability, work instability, and mortality posed by a disease with a global prevalence approaching 20 million.^1^ Recent years have seen a revolution in the management of rheumatoid arthritis, reflecting both a recognition that timely and sustained suppression of inflammation improves outcomes and a growing array of available therapies that target immune cells or cytokine signalling. Such drugs show a similar pattern of efficacy, including ceiling effects, irrespective of their target. Non-response is observed in a third of recipients, and true remission—the absence of clinical signs and symptoms of active rheumatoid arthritis—is uncommon.^2^ Furthermore, some patients with refractory disease have clinically synovitic joint swelling but only grey-scale changes on musculoskeletal ultrasonography without Doppler activity, implying a non-inflammatory pathogenesis to their synovitis. Emerging insights into the molecular events underpinning rheumatoid arthritis increasingly highlight the stromal compartment—specifically, synovial fibroblasts—as crucial drivers of chronicity that have yet to be directly or explicitly targeted in clinical practice. Early in the natural history of the disease, synovial fibroblasts adopt hyperplastic, proliferative phenotypes that perpetuate chronic inflammation or mediate the destruction of cartilage and bone.^3^ Conceivably, synovial fibroblast biology could underpin grey-scale synovitis, as well as the ceiling effects of current targeted therapies. Approaches that modulate synovial fibroblast pathobiology might therefore be required to synergise with current immunomodulatory strategies to reliably induce sustained remission in patients with rheumatoid arthritis.

**Research in context**

**Evidence before this study**

Despite accumulating experimental data supporting a crucial role for synovial fibroblasts as drivers of rheumatoid arthritis, efforts to target them in the clinic remain in their infancy. We searched PubMed, Embase, and ClinicalTrials.gov for studies with abstracts in English published before Nov 20, 2020, for clinical trials targeting synovial fibroblasts in rheumatoid arthritis and adaptive trial designs applied in the condition (see appendix p 1 for search terms and strategy). Three clinical trials of drugs targeting the adhesive properties or crosstalk of synovial fibroblasts via cadherin-11, integrin-α9, and fractalkine were identified; the studies on cadherin-11 and integrin-α9 have reported negative outcomes and efficacy data for fractalkine are awaited. No studies have directly targeted the proliferative capacity of synovial fibroblasts for the treatment of rheumatoid arthritis, via cyclin-dependent kinase (CDK) inhibition or otherwise. Adaptive designs for early phase trials of this kind, although established in oncology, are rare in rheumatic and musculoskeletal diseases; of two such trials in rheumatoid arthritis identified by our search, neither used the Bayesian continual reassessment method.

**Added value of this study**

We found the maximum tolerated dose of seliciclib, an orally available CDK inhibitor, in a phase 1b trial among patients with rheumatoid arthritis refractory to TNF blockade, further evaluating the safety and tolerability of the intervention. We used the Bayesian continual reassessment method, showing it to be efficient for the purposes of dose-finding in this setting. Maximum tolerated dose was derived with a high level of confidence after enrolment of just 15 patients into our trial, considerably fewer than would have been required in a more conventional 3 + 3 design, enabling expeditious clinical development.

**Implications of all the available evidence**

This study paves the way for formal efficacy evaluation of seliciclib to target synovial fibroblast proliferation as a novel strategy in refractory rheumatoid arthritis, and establishes the continual reassessment method as an important adaptive design to consider for future early phase trials in rheumatic and musculoskeletal diseases.

Seliciclib is an orally available cyclin-dependent kinase (CDK) inhibitor under development for oncology indications. Seliciclib suppresses synovial fibroblast proliferation, not only by inhibiting CDK2, but also by inducing expression of the endogenous CDK inhibitor p21, which is otherwise downregulated in synovial fibroblasts in patients with rheumatoid arthritis.^4^, ^5^ In addition, inhibition of CDK7 and CDK9 by seliciclib reduces transcription of the B-cell lymphoma-2 (BCL-2) family member *MCL1*, leading to impaired viability of neutrophils, synovial macrophages, and synovial fibroblasts.^6^ Seliciclib and related CDK inhibitors have shown efficacy and potency in preclinical arthritis models.^7^ Unlike other CDK inhibitors, seliciclib is not myelosuppressive;^8^ its reported toxicity profile is otherwise similar to that of existing conventional synthetic disease-modifying antirheumatic drugs (DMARDs).

These observations, together with evidence from genetic studies that suggest CDK inhibition as a plausible therapeutic strategy in rheumatoid arthritis,^9^ support our hypothesis that seliciclib could be a viable repurposing option for the management of patients with rheumatoid arthritis with disease that is refractory to drugs that target immune inflammation. Our aim was to determine the maximum tolerated dose of seliciclib in patients with active rheumatoid arthritis despite treatment with TNF inhibitors, either as monotherapy or with background conventional synthetic DMARDs. We also aimed to assess safety in this patient group, to assess the relationship between pharmacokinetic and pharmacodynamic parameters and seliciclib toxicity, and to examine any effect on circulating autoantibody status or patient-reported fatigue. Participants' disease activity over time was also evaluated.

## Methods

### Study design and participants

The TRAFIC study is a two part, multicentre, phase 1b/2a trial investigating the safety, tolerability, and potential activity of seliciclib as an addition to biologic therapy in participants with active rheumatoid arthritis; the protocol has been published elsewhere.^10^ Here, we report results of the phase 1b component: a non-randomised, open-label, dose-finding trial done in rheumatology clinics at five UK National Health Service (NHS) hospitals. Adult patients (aged ≥18 years) were eligible for inclusion if they fulfilled the 1987 American College of Rheumatology (ACR) or the 2010 ACR–European League Against Rheumatism classification criteria for rheumatoid arthritis^11^ and had moderate to severe disease activity (defined as a Disease Activity Score for 28 joints [DAS28] of ≥3·2)^12^ despite stable treatment with anti-TNF therapy for at least 3 months before enrolment. Any licensed anti-TNF drug was permissible, either as monotherapy or in combination with methotrexate, sulfasalazine, or hydroxychloroquine (stable doses for ≥4 weeks before the baseline visit). Patients receiving systemic corticosteroid therapy within 4 weeks of the baseline visit, except a stable dose of up to 7·5 mg prednisolone, were ineligible. Full eligibility criteria are shown in the appendix (p 2) and in the protocol.^10^ All participants provided written, informed consent before enrolment into the trial, which received ethical approval from the North East Tyne & Wear South Research Ethics Committee (reference number 14/NE/1075). Patient safety was monitored by an independent data monitoring committee. This trial was registered on Sept 26, 2014, with ISRTN, ISRCTN36667085, and EudraCT, 2014-001339-35.

### Procedures

Eligible patients joined sequentially recruited cohorts of three participants each. Seliciclib was bulk supplied as 200 mg oral capsules by Cyclacel (Dundee, UK); packaging and labelling done by Penn Pharmaceuticals (Tredegar, UK). Participants could receive 200 mg, 400 mg, 600 mg, 800 mg, or 1000 mg, taken daily for 4 consecutive days every week over a 4-week treatment period. Dose range and schedule were predetermined on the basis of healthy control and oncology studies in which more than 450 participants had previously received seliciclib.^10^ In these studies, dose-limiting toxicities were reversible at doses of less than 1600 mg seliciclib daily. Most adverse events were mild to moderate in severity, dose-related, and generally occurred during the first 3 weeks of therapy. Taking into account published^8^, ^13^ and unpublished data from the oncology field, the previous estimate of maximum tolerated dose was 600 mg of seliciclib; to exercise caution, patients enrolled into the first cohort were administered 400 mg, in addition to a background biologic drug as monotherapy or in combination with conventional synthetic DMARD therapy. Seliciclib was dispensed on a weekly basis, with unused capsules retrieved and counted to enable an estimation of compliance, which was cross-checked with patient diaries.

The schedule of study visits has been described elsewhere.^10^ Briefly, regular clinical assessments during the 4-week dosing period included measurement of routine laboratory parameters and an assessment of disease activity (herein we report DAS28–C-reactive protein [CRP]).^12^ Rheumatoid factor and anticitrullinated peptide autoantibodies (ACPA) were measured in local NHS laboratories at baseline and at completion of treatment. Patient reported outcomes, collected after 0, 2, and 4 weeks of treatment with seliciclib, were scored on the 13-item Functional Assessment of Chronic Illness Therapy Fatigue Scale version 4 (FACIT Fatigue)^14^ and the health assessment questionnaire disability index (HAQ-DI).^15^ Seliciclib adherence was assessed by reconciliation of the number of tablets dispensed and returned. Adverse events attributed as definitely, probably, or possibly related to seliciclib, as well as those considered unrelated, were categorised as mild, moderate, or severe on the basis of symptoms or laboratory parameters and according to previously described criteria.^10^ Comprehensive safety monitoring, including the documentation of serious adverse events and seliciclib-related serious adverse events, was done according to International Conference on Harmonisation Good Clinical Practice principles, and the expectedness of incident seliciclib-related adverse events was determined with reference to seliciclib's investigator brochure.

The terminal half-life of seliciclib is 3–4 h, as the primary route of metabolism is via cytochromes 3A4 and 2B6.^13^, ^16^ To determine participant-level pharmacokinetics, blood samples were drawn at 0, 2, 4, and 6 h after administration of seliciclib on day 1 of week 1 and week 4; drug concentrations were measured using a validated liquid chromatography-tandem mass spectrometry method. Following calculation of the maximum concentration (C_max_) and area under the plasma concentration-time curve 0–6 h (AUC_0–6_) of seliciclib, the relationship of these parameters with safety and tolerability in the overall trial population was assessed. AUC_0–6_ was calculated using a non-compartmental trapezoidal estimate.^17^ Based on data from the oncology setting, determination of a maximum tolerated dose of 200 mg in this phase 1b trial using a continual reassessment method would mandate demonstration of a pharmacodynamic effect at that dose to justify ongoing clinical development (Frame S, unpublished). Therefore, at the same time that blood was drawn for pharmacokinetics, blood was also drawn into TEMPUS tubes (ThermoFisher Scientific, Waltham, MA, USA) for RNA stabilisation to enable whole blood measurement of the *MCL1* gene if required, with decreased expression expected early following target engagement by seliciclib via selective CDK7-mediated or CDK9-mediated inhibition.

### Outcomes

The primary objective of the phase 1b component of the study was identification of the maximum tolerated dose of seliciclib in patients with active rheumatoid arthritis despite treatment with anti-TNF drugs as either monotherapy or with background conventional synthetic DMARDs. The secondary objectives were to assess the tolerability and safety of seliciclib; to assess the relationship of pharmacokinetic and pharmacodynamic biomarkers with toxicity; to assess any effect of seliciclib on fatigue associated with rheumatoid arthritis; and to assess any effect of seliciclib on rheumatoid arthritis-associated autoantibody status. DAS28–CRP data were used in a post-hoc exploratory analysis of disease activity over time.

### Statistical analysis

We used a restricted, one-stage Bayesian continual reassessment method^18^ to determine the maximum tolerated dose for seliciclib (200 mg, 400 mg, 600 mg, 800 mg, or 1000 mg) over the 4-week treatment window based on a target dose-limiting toxicity rate of 35%. This a priori rate was chosen because it was similar to that seen for established conventional synthetic DMARDs, including methotrexate.^19^ The continual reassessment method is a model-based design that has been shown to be more accurate and potentially more efficient in identifying the maximum tolerated dose than traditional rule-based approaches such as the 3 + 3 design.^20^ A dose-limiting toxicity was defined as an adverse event or adverse reaction that occurred during the treatment period resulting in a participant's request to withdraw or laboratory parameter derangement beyond predefined cutoffs, and leading to cessation of seliciclib.^10^ In the event of several adverse events or adverse reactions contributing to the decision to discontinue seliciclib, only a single dose-limiting toxicity was recorded for purposes of the continual reassessment method. Using a one-parameter logistic continual reassessment model, initial estimates of the probability of dose-limiting toxicity at each dose (the skeleton) were chosen via Monte Carlo simulation, evaluating objective performance measures under clinically relevant scenarios to yield a probability of 0·13 at 200 mg, 0·23 at 400 mg, 0·35 at 600 mg, 0·47 at 800 mg, and 0·58 at 1000 mg. The prior standard deviation of the single model parameter was 0·27. Subject to approval by the sponsor and the data monitoring committee, each subsequent cohort's dose was determined algorithmically according to the continual reassessment method using cumulative toxicity data, until the maximum tolerated dose was established. Planned enrolment was for a maximum of 21 patients, as seven cohorts of three patients each. Early termination of recruitment was possible at the discretion of the data monitoring committee and independent trial steering committee according to prespecified stopping rules: (1) if sufficient patients had been allocated to justify the current maximum tolerated dose estimate, and the same dose would be recommended for a subsequent cohort were the trial to continue; or (2) if the lowest dose (200 mg) was considered too toxic—ie, there was a high probability (>0·7) that the posterior probability of dose-limiting toxicity at the lowest dose was greater than the target dose-limiting toxicity rate. A full description and justification of the trial's statistical design has been submitted for publication and is available from the authors on reasonable request.

### Role of the funding source

Cyclacel were involved in discussions regarding study design, contributed the study drug, funded measurement of pharmacokinetics and pharmacodynamics, reviewed and approved the report, and had a role in data collection, data interpretation, and writing of the report. The funder had no role in data analysis.

## Results

**Figure 1:**
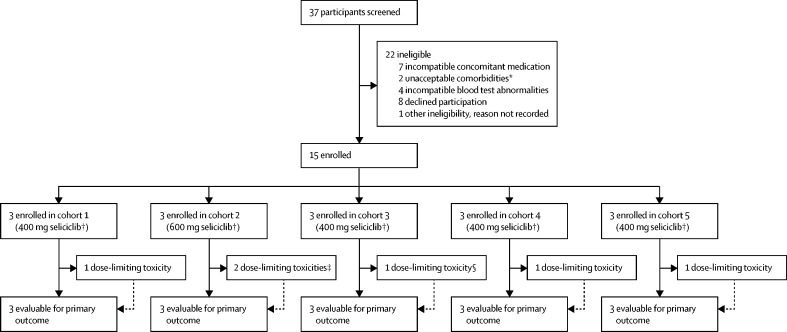
Trial profile All enrolled participants contributed evaluable data, no participants were lost to follow-up. *Comorbidities were determined to be unacceptable for trial entry at the discretion of the investigator in line with the trial protocol. †Seliciclib was taken daily for 4 consecutive days every week over a 4 week treatment period. ‡One patient had a serious adverse event: fever and acute kidney injury. §One patient had a serious adverse event: liver injury.

Between Oct 8, 2015, and Aug 15, 2017, 37 patients were screened and 15 were enrolled and received seliciclib (figure 1). 22 patients were defined as ineligible at screening or, in one case, at baseline, before treatment. Although enrolment of up to seven cohorts of three participants each (21 patients in total) was specified in the design, the primary analysis for the trial was event-driven, permitting early termination of enrolment under the prespecified stopping rules as described.^10^ After treatment of five cohorts, the trial steering committee, in consultation with the data monitoring committee, determined that a sufficient number of patients had been treated to define the maximum tolerated dose with confidence. Enrolment to the phase 1b component of the trial was therefore concluded after 15 participants, emphasising the potential efficiency of such a Bayesian design. Participant baseline characteristics are shown in table 1. Documented seliciclib tablet reconciliation suggested 100% compliance among all trial participants with prescribed dosing during the course of the study.

**Table 1:** Participant characteristics

		**Participants (n=15)**
Age, years		58 (39–76)
Sex		
	Female	13 (87%)
	Male	2 (13%)
White British		15 (100%)
Disease duration, years		8 (3–30)
Swollen joint count		4 (0–11)
Tender joint count		10 (1–26)
DAS28–CRP score		4·9 (3·5–6·3)
Erythrocyte sedimentation rate, mm/h		9 (2–72)
CRP, mg/L		4 (1–69)
Concomitant anti-TNF therapy*		
	Etanercept	6 (40%)
	Adalimumab	4 (27%)
	Certolizumab	2 (13%)
	Golimumab	3 (20%)
Concomitant csDMARDs		
	None†	5 (33%)
	Methotrexate	4 (27%)
	Hydroxychloroquine	1 (7%)
	Methotrexate plus sulfasalazine	1 (7%)
	Methotrexate plus hydroxychloroquine	2 (13%)
	Sulfasalazine plus hydroxychloroquine	1 (7%)
	Methotrexate plus sulfasalazine plus hydroxychloroquine	1 (7%)

Data are median (range) or n (%). CRP=C-reactive protein. csDMARDs=conventional synthetic disease-modifying antirheumatic drugs. DAS28–CRP=Disease Activity Score for 28 joints–C-reactive protein.

* Originator or biosimilar agents permitted.

† Of the recipients of anti-TNF monotherapy at baseline, two received etanercept, two certolizumab, and one golimumab.

**Figure 2:**
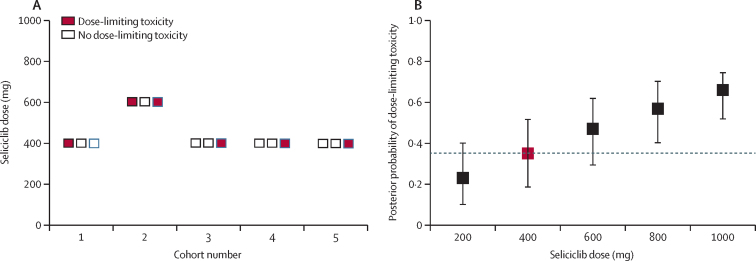
Dose-limiting toxicity occurrence by cohort and dose and Bayesian posterior probability of dose-limiting toxicity at each dose (A) Dose limiting toxicity occurrence by cohort and dose level. Each box represents one patient. (B) Bayesian posterior probability of dose-limiting toxicity at each dose level (with 90% posterior probability intervals) following completion of cohort five. Dashed line at posterior probability of dose-limiting toxicity of 0·35; red point indicates value closest to target.

The seliciclib starting dose for cohort one was 400 mg. Application of the continual reassessment algorithm prompted a single dose increment to 600 mg for cohort two, but reversion to 400 mg for subsequent cohorts (Figure 1, Figure 2). Before enrolment of cohort six, four cohorts had received 400 mg seliciclib, with dose-limiting toxicity observed in one (33%) of three patients in each cohort, including in three consecutive cohorts. At this stage, the probability that the maximum tolerated dose would change after completion of cohort six was estimated to be 0·04 and, in consultation with the data monitoring committee, it was therefore considered sufficiently well established and recruitment was stopped after completion of cohort five. Hence, the maximum tolerated dose, the dose closest to that at which 35% of patients have dose-limiting toxicity, was 400 mg. The Bayesian posterior probability of dose-limiting toxicity at 400 mg seliciclib is 0·35 (90% posterior probability interval 0·18–0·52; figure 2B). Previous and Bayesian posterior probabilities of dose-limiting toxicity after each cohort's completion are shown in the appendix (p 7).

Six (40%) of 15 patients had dose-limiting toxicity during the course of the trial (Figure 1, Figure 2), all of which occurred within the first two treatment cycles (table 2). Symptoms of nausea and vomiting contributed to all but one of the dose-limiting toxicities (five [83%] of six, including both of those occurring at the 600 mg dose). Two serious adverse events occurred during the trial, both were considered to be related to seliciclib and contributed to two separate dose-limiting toxicities (figure 1; table 2). One participant (cohort two) described overnight symptoms of fever and gastrointestinal upset (nausea and vomiting) after a single dose of 600 mg seliciclib. Despite symptomatic improvement on hospital assessment the following day, this was found to be associated with acute kidney injury (peak serum creatinine concentration 203 μmol/L), and seliciclib was discontinued in this participant; renal function normalised by day 8. Another participant (cohort three) had a more insidious onset of gastrointestinal symptoms (constipation, nausea, and vomiting), which became associated with drug-induced liver injury including clinical jaundice after eight doses of 400 mg seliciclib (peak alanine aminotransferase concentration 667 U/L; peak bilirubin concentration 53 μmol/L); seliciclib was discontinued, with complete normalisation of liver function tests and symptom resolution confirmed after 7 days. Neither serious adverse event was unexpected based on the investigator brochure for seliciclib, and there were no deaths. No patients withdrew from the trial early due to uncontrolled rheumatoid arthritis symptoms, although three patients required intra-articular injections of corticosteroid as rescue therapy for rheumatoid arthritis, as permitted by the trial protocol.

**Table 2:** Summary of dose-limiting toxicities

	**Cohort**	**Age, years**	**Sex**	**Seliciclib dose, mg**	**Number of doses received**	**Background anti-TNF**	**Concomitant csDMARDs**	**Number of contributing adverse events**	**Number of contributing serious adverse events**	**Description of dose-limiting toxicity**	Outcome*
Patient 1	One	47	Female	400 mg	8	Etanercept	..	3	0	Constipation, nausea, vomiting, abnormal liver enzymes, fatigue	Resolved
Patient 4	Two	52	Female	600 mg	4	Certolizumab	..	3	0	Constipation, nausea, vomiting	Resolved
Patient 6	Two	64	Male	600 mg	1	Etanercept	..	0	1†	Fever, nausea, vomiting, acute renal injury	Resolved
Patient 9	Three	52	Female	400 mg	8	Adalimumab	Sulfasalazine, hydroxychloroquine	3	1†	Constipation, nausea, vomiting, jaundice, abnormal liver enzymes, abnormal bilirubin	Resolved
Patient 12	Four	39	Female	400 mg	8	Golimumab	Methotrexate	4	0	Fever, dizziness, abnormal liver enzymes	Resolved
Patient 15	Five	61	Female	400 mg	8	Certolizumab	..	7	0	Dizziness, nausea, vomiting, abnormal liver enzymes, abnormal bilirubin	Declining liver enzymes

csDMARDs=conventional synthetic disease-modifying antirheumatic drugs.

* Outcome of contributory adverse event or serious adverse event at close of follow-up.

† Serious adverse events were classified as expected based on the investigator brochure.

65 non-serious adverse events were recorded during the trial, and 50 of these were defined as treatment related, being considered definitely, probably, or possibly caused by seliciclib (table 3). 20 of the treatment-related, non-serious adverse events contributed to dose-limiting toxicities (table 2; details of all non-serious adverse events for all participants, including their documented causal relationship with seliciclib and contribution to dose-limiting toxicities, are in the appendix pp 3–5). Treatment-related adverse events affecting the gastrointestinal system or liver function were common, with nausea being the most frequently reported treatment-related adverse event (14 [28%] of 50 adverse events). 12 (67%) of 18 terms used to describe treatment-related adverse events indicated gastrointestinal or liver involvement, comprising 38 (76%) of 50 treatment-related adverse events. The majority of adverse events at both seliciclib doses were mild, and all adverse events suspected to be related to treatment had resolved by the end of the trial, with the exception of two viral respiratory tract infections (which subsequently resolved) and one persistent elevation in aspartate aminotransferase concentration (3·5 times the upper limit of normal), which was nonetheless declining 2 weeks after seliciclib cessation. Adverse events, serious adverse events, and dose-limiting toxicities did not differ in frequency between the ten (67%) participants also taking background concomitant conventional synthetic DMARDs and the five (33%) on anti-TNF monotherapy. No request by a patient to stop treatment during the study followed an event considered unrelated to seliciclib by the investigating team.

**Table 3:** Treatment-related adverse events

	**400 mg seliciclib dose (41 adverse events in 12 participants)**			**600 mg seliciclib dose (9 adverse events in 3 participants)**			**Total treatment-related adverse events (% of total)**
	Mild	Moderate	Severe	Mild	Moderate	Severe	
Nausea	5	3	2	3	0	1	14 (28%)
Increased ALT	3	2	1	0	0	0	6 (12%)
Fatigue	2	2	1	0	0	0	5 (10%)
Diarrhoea	1	0	1	0	0	1	3 (6%)
Abdominal pain	0	0	1	2	0	0	3 (6%)
Increased AST	2	1	0	0	0	0	3 (6%)
Dizziness	2	0	0	0	0	1	3 (6%)
Increased ALP	2	0	0	0	0	0	2 (4%)
Heartburn	2	0	0	0	0	0	2 (4%)
Anorexia	1	0	0	0	0	0	1 (2%)
Increased bilirubin	1	0	0	0	0	0	1 (2%)
Flatulence	1	0	0	0	0	0	1 (2%)
Fever	1	0	0	0	0	0	1 (2%)
Vomiting	0	1	0	0	0	0	1 (2%)
Rhinitis	0	0	0	1	0	0	1 (2%)
Viral upper respiratory tract infection	1	0	0	0	0	0	1 (2%)
Viral lower respiratory tract infection	1	0	0	0	0	0	1 (2%)
Jaundice	0	0	1	0	0	0	1 (2%)

The terms fatigue and sleepiness are concatenated for purposes of reporting; as are heartburn and indigestion. ALP=alkaline phosphatase. ALT=alanine aminotransferase. AST=aspartate aminotransferase.

Plasma seliciclib AUCs and C_max_ measurements at 0, 2, 4, and 6 h following seliciclib ingestion on day 1 and (where available) day 28 are shown in the appendix (pp 6, 8). As expected, AUC_0–6_ and C_max_ values on day 1 of the first treatment cycle were higher among the three recipients of the 600 mg seliciclib dose in cohort two (AUC_0–6_ median 5753 ng/mL per h and C_max_ 1560 ng/mL) than in the participants on the lower dose (AUC_0–6_ median 2004 ng/mL per h and C_max_ 655 ng/mL). Patients who subsequently had a dose-limiting toxicity had a higher median seliciclib AUC_0–6_ (4484 ng/mL per h) and C_max_ (1249 ng/mL) than patients who did not have a dose-limiting toxicity (median AUC_0–6_ 2090 ng/mL per h and C_max_ 637 ng/mL). The results were similar among patients who received the 400 mg seliciclib dose (ie, excluding cohort two), with the median AUC_0–6_ and C_max_ values being approximately twice as high in patients who had dose-limiting toxicities compared with those who did not (results shown in full in the appendix p 6). Our sample size did not support formal inference testing, and the pharmacokinetic values for the two recipients of the 400 mg dose who had serious adverse events fell within the relevant interquartile ranges. Nonetheless, these data raise the possibility of a relationship between toxicity and pharmacokinetic parameters that warrants further scrutiny in downstream studies. AUC_0–6_ and C_max_ values were similar at day 1 and day 22 in patients for whom measurements at both timepoints were available. Pharmacodynamic parameters were not investigated because no dose reduction to 200 mg was triggered by the continual reassessment algorithm.

**Figure 3:**
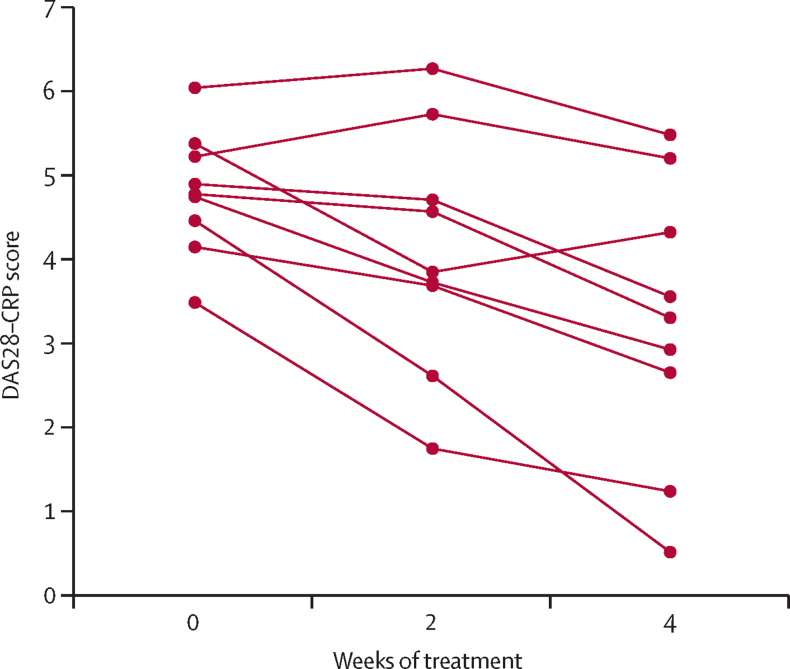
DAS28–CRP scores before seliciclib and up to 4 weeks of treatment among patients who completed treatment Each line shows the scores for one patient. DAS28–CRP=Disease Activity Score for 28 joints–C-reactive protein.

Among participants who were seropositive for rheumatoid factors, ACPA, or both on day 1 of seliciclib treatment, no change in measured autoantibody concentrations (measured as IU for rheumatoid factors and U/mL for ACPA) or status (positive *vs* negative according to local laboratory cutoffs) were observed during the trial (data not shown). Disease activity (DAS28–CRP) was recorded at 0, 2, and 4 weeks for nine (60%) of 15 participants who completed treatment. In a post-hoc analysis, a median change in DAS28–CRP score of −1·5 (range 0 to −4) was observed from week 0 to week 4 in these patients (figure 3; participant-level data for all 15 participants are shown in appendix p 9). Although fatigue was the descriptor for five adverse events (table 3), FACIT Fatigue scores (available for nine [60%] patients) showed no substantial change during the 4-week treatment course (score increased from median 27·0 [range 16 to 39] to 33·0 [17 to 51], indicating a diminution in fatigue; appendix p 10). HAQ-DI scores remained broadly stable during the trial (data not shown).

## Discussion

This study identified a maximum tolerated dose of 400 mg seliciclib in patients with active rheumatoid arthritis refractory to anti-TNF therapy, either as monotherapy or with background conventional synthetic DMARDs. To our knowledge, this is the first reported clinical trial expressly intended to target the hyperplastic, proliferative properties of synovial fibroblasts in rheumatoid arthritis, and the first rheumatology trial to use the Bayesian continual reassessment method. Expansion of the synovial membrane into a pannus tissue, which actively erodes cartilage and bone while sustaining local inflammation, is a hallmark of rheumatoid arthritis; the process is dependent on the adoption by synovial fibroblasts of a proliferative and apoptosis-resistant phenotype. Our strategy of repurposing a CDK inhibitor to limit synovial fibroblast cell cycle progression affords an efficient development pipeline for drugs that, in reversing the quasi-malignant properties of these cells, might overcome ceiling effects of drugs targeting immune inflammation alone.

A few studies have targeted other properties of synovial fibroblasts, although examples in clinical development are rare. Efforts to disrupt the synovial membrane's intrinsic cohesiveness during pannus development via cadherin-11 or integrin-α9—adhesion molecules that are expressed by synovial fibroblasts—have hitherto been unsuccessful.^21^, ^22^, ^23^ A study seeking to inhibit leukocyte chemotaxis by, and cross-talk with, synovial fibroblasts by targeting the synovial membrane-expressed chemokine CX3CL 1 (fractalkine) is ongoing.^24^ Our strategy might have parallel advantages, both restraining tissue-degrading proteinase expression and inhibiting pathological neutrophil and synovial macrophage function.^25^, ^26^, ^27^ The results of the phase 1b trial reported here are a crucial precursor to an ongoing phase 2a efficacy evaluation that is testing these hypotheses.

Application of the continual reassessment model approach in our dose-finding evaluation was efficient, with the maximum tolerated dose determined to a high confidence level after enrolment of 15 out of the permitted target of 21 participants (five cohorts of a possible seven). This illustrates an attractive feature of the adaptive Bayesian design, whereby progression of a drug with acceptable safety and tolerability parameters is not delayed by enrolment of analytically redundant participants in a more typical 3 + 3 phase 1 trial design, with the associated ethical and financial implications. Rather, such participants can be offered expedited enrolment into the follow-on phase 2a trial. The application of adaptive trial designs in rheumatology is in its infancy^28^, ^29^ but, given well documented challenges of recruitment to early phase clinical trials,^30^ our approach sets a valuable precedent and basis for the investigation of experimental investigational medicinal products for rheumatoid arthritis in the future.

Early phase oncology clinical trials with dosing schedules similar to that used in the current investigation indicated doses of 800 mg or more of seliciclib were safe and broadly acceptable to patients, albeit typically administered in divided daily doses.^14^ The somewhat lower, once-daily maximum tolerated dose of 400 mg observed in our study might reflect a reduced tolerability threshold among patients with rheumatoid arthritis with moderately active disease compared with patients with cancer diagnoses refractory to alternative therapy. This highlights the importance of recalibrating a drug's posology for each new clinical indication. Such considerations are not new, with the anchor conventional synthetic DMARD methotrexate licensed for rheumatological indications at only a fraction of the dose used in haematological malignancies for which it was originally developed. The median plasma C_max_ and AUC_0–6_ values for seliciclib among 12 recipients of the 400 mg dose are compatible with pharmacological activity (Frame S, unpublished), and synovial tissue pharmacodynamics form an important component of our phase 2a study.

Adverse events and dose-limiting toxicities in the current clinical trial were similar to the range of toxicities observed in oncology, with gastrointestinal reactions including liver enzyme abnormalities being the most common among these. This range of side-effects is familiar to rheumatologists and their patients, for whom nausea, anorexia, transaminitis, and fatigue are commonly associated with conventional synthetic DMARD use. A more generalised systemic upset, accompanied by fever and transient kidney injury as occurred in one recipient of the higher seliciclib dose in our study, is more rarely observed with licensed therapies, although it might be an idiosyncratic consequence of sulfasalazine use, for example. An increase in dose-limiting toxicity rate was not observed when seliciclib was given in combination with background conventional synthetic DMARDs. On the contrary, four of five patients receiving anti-TNF monotherapy had a dose-limiting toxicity, whereas eight of ten patients who were receiving anti-TNF drugs and concomitant conventional synthetic DMARDs did not. Caution should be exercised when considering this small sample size, but with anti-TNF monotherapy identifying a patient subgroup intolerant of multiple conventional synthetic DMARDs under current treatment guidelines,^31^ our observation seems to be consistent with clinical experience of a poorly understood phenomenon of treatment intolerance in these patients. Similarly, the small sample size makes it difficult to confirm or exclude a relationship between pharmacokinetic parameters and toxicity, although our finding that patients with dose-limiting toxicities had median seliciclib AUC_0–6_ and C_max_ values that were twice as high on day 1 of treatment cycle 1 compared with patients without dose-limiting toxicities suggests hypotheses to test in future studies. Such studies should also incorporate an assessment of pharmacodynamic parameters in relation to both efficacy and toxicity.

This uncontrolled, phase 1a trial was not designed to assess efficacy, and the reduction of median DAS28–CRP observed among nine patients who completed treatment might represent regression to the mean, but no detrimental effect of the intervention on disease activity was suggested. Overall, although careful pharmacovigilance will be a feature of phase 2 seliciclib studies for rheumatoid arthritis, no impediment to their progression on safety grounds has been identified.

## Data sharing

Due to commercial sensitivity, the data beyond those presented in the manuscript and appendix are not immediately available for sharing. Should this status change, requests will be considered by the research team, including Newcastle Clinical Trials Unit and the Chief Investigator, in the context of the research question proposed, as well as ethical considerations such as patient anonymity and informed consent. The patient information sheet and informed consent documentation are available on request from the corresponding author. The clinical trial protocol has been published previously.^10^
