# Supplementary material for: Targeting synovial fibroblast proliferation in rheumatoid arthritis (TRAFIC): an open-label, dose-finding, phase 1b trial
Source: Lancet Rheumatol. 2021 Mar 9;3(5):e337–46. doi: 10.1016/S2665-9913(21)00061-8 (PMC8062952; doi:10.1016/S2665-9913(21)00061-8)
Supplement: Supplementary appendix [file mmc1.pdf]

THE LANCET
Rheumatology

Supplementary appendix

This appendix formed part of the original submission and has been peer reviewed. We post it as supplied by the authors.

Supplement to: Pratt A G, Siebert S, Cole M, et al. Targeting synovial fibroblast proliferation in rheumatoid arthritis (TRAFIC): an open-label, dose-finding, phase 1b trial. *Lancet Rheumatol* 2021; published online March 9. https://doi.org/10.1016/S2665-9913(21)00061-8.

Targeting synovial fibroblast proliferation in rheumatoid arthritis: a phase 1b trial.

Pratt AG *et al*.

Supplementary File.

Research in Context Search Terms and Strategy.

Two search terms were employed to underpin the content of the Research in Context section, as below. In each case, results were reviewed manually to identify relevant items.

1. Trials targeting the synovial fibroblast in Rheumatoid Arthritis.

(“Fibroblast” OR (((“cyclin dependent kinase” OR “CDK”) AND “inhibitor”) OR “CDKI”)) AND “Rheumatoid Arthritis” AND “trial”.

Three relevant interventional clinical trials were identified, included in the manuscript as references 20, 21 and 22.

2. Adaptive designs in rheumatoid arthritis clinical trials.

(“Rheumatoid arthritis” OR “RA”) AND (“adaptive design” OR "adaptive trial design" OR "Bayesian adaptive" OR (“continuous reassessment method” OR “CRM”)).

Three relevant original articles describing clinical trials in rheumatoid arthritis that adopted adaptive designs were identified, included in the manuscript as references 27 and 28; neither of these specifically employed the continuous reassessment method (CRM) used in our investigation.

Supplementary Tables.

Table S1

Inclusion criteria (participants must fulfil all of the following):
Rheumatoid arthritis fulfilling the 1987 ACR or 2010 ACR/EULAR criteria
Age ≥ 18 years
At least 6 months' disease duration
ACR Functional Class I-III
DAS28 ≥3.2
Currently taking anti-TNF as part of standard clinical care, and have received stable treatment with a single anti-TNF agent for at least 3 months upon entry to the study.
Anti-TNF may be administered as either monotherapy or with background conventional DMARDs. Permitted background DMARDs are methotrexate, sulphasalazine and hydroxychloroquine, either alone or in combination, at stable dose(s) for ≥4 weeks prior to baseline visit.
No intramuscular glucocorticoid administration in the 4 week period prior to baseline visit. Stable dose of non-steroidal anti-inflammatory drug (NSAID) or corticosteroid (prednisolone≤7.5 mg) for ≥4 weeks as part of standard clinical care are allowed.
**Exclusion criteria (participants must not have any of the following):**
If patients were previously taking leflunomide a minimum period of 20 days must have elapsed between the last dose of leflunomide and the first dose of IMP.
Use of other investigational medicinal products within 30 days prior to trial entry (defined as date of recruitment into trial).
Serious or unstable co-morbidity deemed unsuitable by principal investigator e.g. COPD, cardiac failure, other significant autoimmune disease
Patients must not drink more than 2 units of alcohol per day and no more than 10 units of alcohol per week during the trial and for a 4 week period after completion of the trial.
Known active infection at screening visit or at baseline (except fungal nail infection)
Infection requiring hospitalization or IV antibiotics within 6 weeks prior to baseline
History of recurrent or chronic infection
Recent live vaccination (within 6 weeks of baseline)
Haemoglobin <10g/dL; neutrophils< 1.5 x10^9/L; platelets <100x10^9/L
Patients taking ketoconazole, voriconazole, erythromycin, clarithromycin.
ALT or ALP>1.5x upper limit of normal
Estimated glomerular filtration rate <60ml/minute
Major surgery within 8 weeks prior to baseline or planned within 3 months from baseline.
Pregnancy, or women planning to become pregnant within the trial period, or women who are breast feeding
Females or males of child-bearing potential unwilling to use two forms of adequate contraception whilst taking the IMP and for one month afterwards.

Supplementary Table S1*. Full list of inclusion criteria for part 1 of TRAFIC study.*

Table S2

Subject	Cohort	Seliciclib dose (mg)	Adverse Event	Relationship to treatment[A]	Severity[B]	Contribution to DLT	Resolved at end of trial
1	1	400	Nausea	Probably	Moderate	No	Yes
			Fatigue	Probably	Moderate	No	Yes
			Heartburn	Probably	Mild	No	Yes
			Anorexia	Probably	Mild	No	Yes
			Nausea	Probably	Severe	**Yes**	Yes
			Fatigue	Probably	Severe	**Yes**	Yes
			AST increased	Probably	Moderate	**Yes**	Yes
			ALT increased	Probably	Mild	No	Yes
			Blood ALP increased	Probably	Mild	No	Yes
2	1	400	Nausea	Definitely	Moderate	No	Yes
			Nausea	Definitely	Moderate	No	Yes
			Shingles on forehead	Unrelated	Moderate	No	Ongoing
3	1	400	Sleepiness	Probably	Moderate	No	Yes
			Sleepiness	Probably	Mild	No	Yes
			Sleepiness	Probably	Mild	No	Yes
			Nausea	Possibly	Mild	No	Yes
			Flatulence	Possibly	Mild	No	Yes
4	2	600	Nausea	Definitely	Severe	**Yes**	Yes
			Dizziness	Definitely	Severe	**Yes**	Yes
			Diarrhoea	Definitely	Severe	**Yes**	Yes
5	2	600	Nausea	Possibly	Mild	No	Yes
			Nausea	Possibly	Mild	No	Yes
			Nausea	Possibly	Mild	No	Yes
			Stomach pain	Probably	Mild	No	Yes

			Runny nose	Possibly	Mild	No	Yes
			Flaking finger nails	Unrelated	Mild	No	Ongoing
			Stomach pain	Probably	Mild	No	Yes
6[C]	2	600	Immunoglobulin monoclonal gammopathy of undetermined significance	Unrelated	Mild	No	Ongoing
7	3	400	Dermatitis on both hands	Unlikely	Mild	No	Yes
			Low mood	Unlikely	Mild	No	Yes
8	3	400	Nausea	Probably	Mild	No	Yes
			Indigestion	Possibly	Mild	No	Yes
9[C]	3	400	Nausea	Probably	Mild	No	Yes
			Diarrhoea	Probably	Mild	No	Yes
			Nausea	Probably	Severe	**Yes**	Yes
			Diarrhoea	Probably	Severe	**Yes**	Yes
			Abdominal pain	Probably	Severe	**Yes**	Yes
10	4	400	Leukocytes in urine	Unlikely	Mild	No	Yes
			Leukocytes in Urine	Unlikely	Mild	No	Ongoing
			Coryzal symptoms	Unlikely	Mild	No	Yes
			Right side of neck - enlarged lymph nodes	Unlikely	Mild	No	Yes
			Urinary tract infection - E Coli	Unlikely	Mild	No	Yes
			Chest Infection - Sputum culture Moraxella Catarrhalis	Unlikely	Mild	No	Yes
11	4	400	Nausea	Possibly	Mild	No	Yes
			ALT increased	Possibly	Mild	No	Yes
			URTI (viral)	Possibly	Mild	No	Ongoing
			LRTI (viral)	Possibly	Mild	No	Ongoing
			Cough	Unrelated	Mild	No	Ongoing
12	4	400	ALT increased	Probably	Mild	**Yes**	Yes
			AST increased	Probably	Mild	**Yes**	Yes
			Dizziness	Probably	Mild	**Yes**	Yes

			Fever	Probably	Mild	**Yes**	Yes
13	5	400	Headache	Unlikely	Mild	No	Yes
14	5	400	ALT increased	Possibly	Moderate	No	Yes
			Right knee soft tissue injury	Unrelated	Moderate	No	Ongoing
			Transient ear ache	Unlikely	Mild	No	Yes
15	5	400	Nausea	Definitely	Mild	**Yes**	Yes
			Vomiting	Definitely	Moderate	**Yes**	Yes
			ALT increased	Definitely	Severe	**Yes**	Yes
			AST increased	Definitely	Mild	**Yes**	Ongoing
			Blood alkaline phosphatase increased	Definitely	Mild	**Yes**	Yes
			Dizziness	Possibly	Mild	**Yes**	Yes
			Jaundice	Definitely	Severe	No	Yes
			ALT increased	Definitely	Moderate	No	Yes
			Bilirubin increase	Definitely	Mild	**Yes**	Yes

Supplementary Table S2*. Line listing of all non-serious adverse events. ALP: alkaline phosphatase; AST: aspartate aminotransferase; ALT: alanine transaminase; DLT: Dose-limiting toxicity; U/LRTI: upper/lower respiratory tract infection [A]Causality classified by supervising investigator, with terms 'definitely,' 'probably' or 'possibly' being required to define a treatment related AE, as summarised in Table 2 of main manuscript; [B]severity criteria classified as described in Reference 3. [C]Patients 6 and 9 both experienced SAEs. Identifiers underlined for subjects who experienced a DLT.*

Table S3

Patient ID	Cohort number	Protocol Dose (mg)	Percentage of protocol dose received		Seliciclib $AUC_{0\text{-}6hr}$ (ng/ml.hr)		Seliciclib C_{max} (ng/ml)		Number of related (possibly, probably, definitely) AEs[A]	Number of related (possibly, probably, definitely) SAEs
			Week 1	Week 4	Day 1 Week 1	Day 1 Week 4	Day 1 Week 1	Day 1 Week 4		
**1**	1	400	100	**NA**	5875	NA	1570	NA	9	0
**2**	1	400	100	100	717	597	443	188	2	0
**3**	1	400	100	100	483	363	165	110	5	0
**4**	2	600	100	NA	5753	NA	1560	NA	3	0
**5**	2	600	100	100	7217	1182	2640	461	6	0
**6**[B]	2	600	25	NA	3605	NA	937	NA	0	1
**7**	3	400	100	125	2090	1688	775	670	0	0
**8**	3	400	125	100	5127	5498	1070	1700	2	0
**9**[B]	3	400	100	NA	1757	NA	678	NA	5	1
**10**	4	400	100	100	2619	2190	632	625	0	0
**11**	4	400	100	100	2179	1409	720	534	4	0
**12**	4	400	100	NA	5362	NA	1680	NA	4	0
**13**	5	400	100	100	1919	1807	478	539	0	0
**14**	5	400	125	100	1045	1320	257	311	1	0
**15**	5	400	100	NA	1373	NA	319	NA	9	0

Supplementary Table S3. *Seliciclib dosing, seliciclib pharmacokinetic parameters and related adverse events by patient.* [A]*There were 50 related (possibly, probably, definitely) AEs in total of which 31 were mild, 9 moderate and 10 severe.* [B]*Patients 6 and 9 both experienced SAEs. Identifiers underlined for subjects who experienced a DLT.*

Supplementary Figures.

Figure S1.

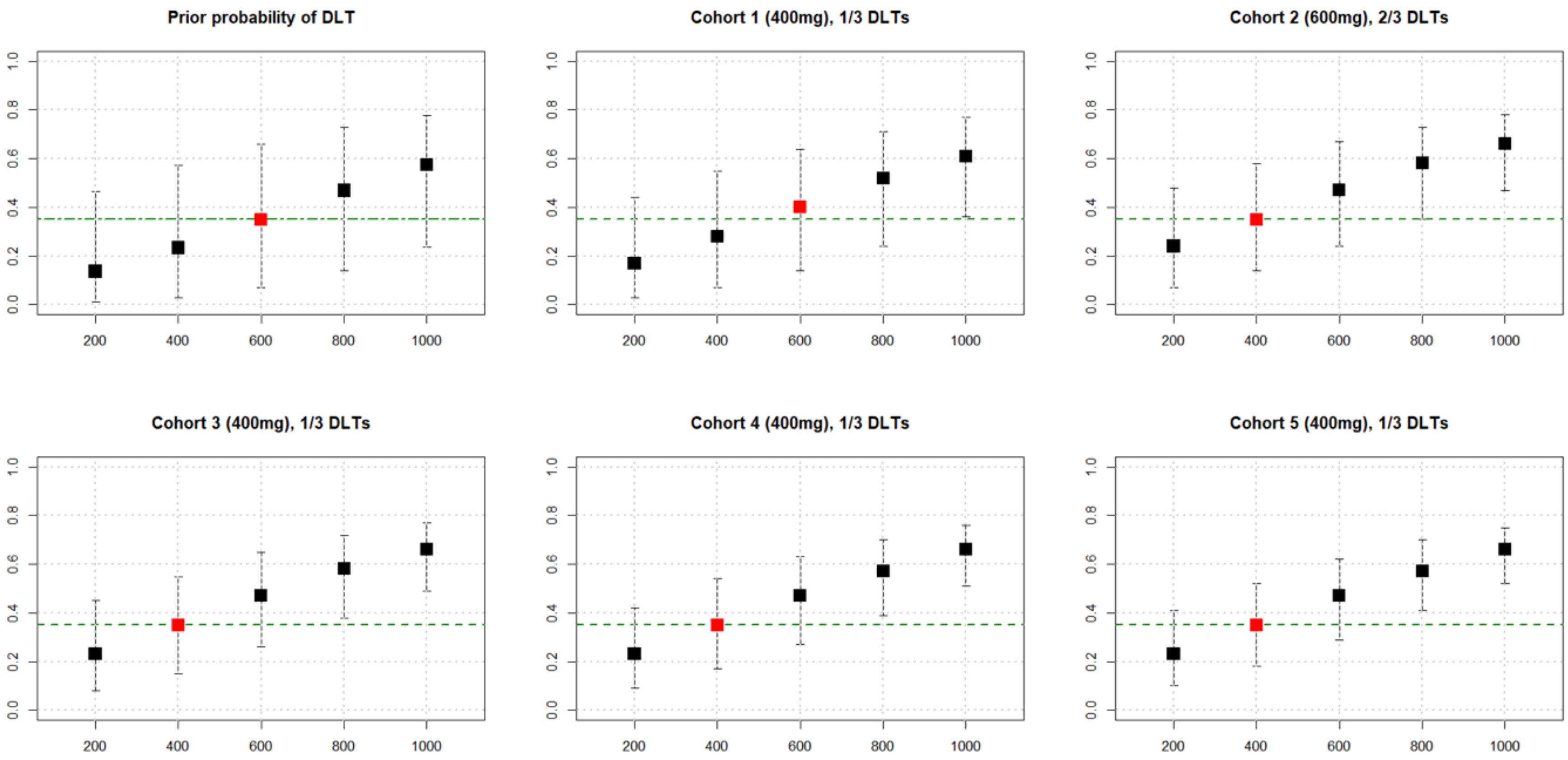

Figure S1. *The prior probability of dose limiting toxicity (DLT; y axis) at each dose level (x axis) is depicted in the top left panel; error bars indicate probability intervals. In remaining panels, Bayesian posterior probabilities of DLT after completion of the indicated cohort (with its dosing level and DLT rate) are depicted. Estimated maximum tolerated dose (MTD) is indicated in red at each stage, and does not change after completion of cohort 2, but the probability intervals can be seen to narrow as the trial progresses.*

Figure S2.

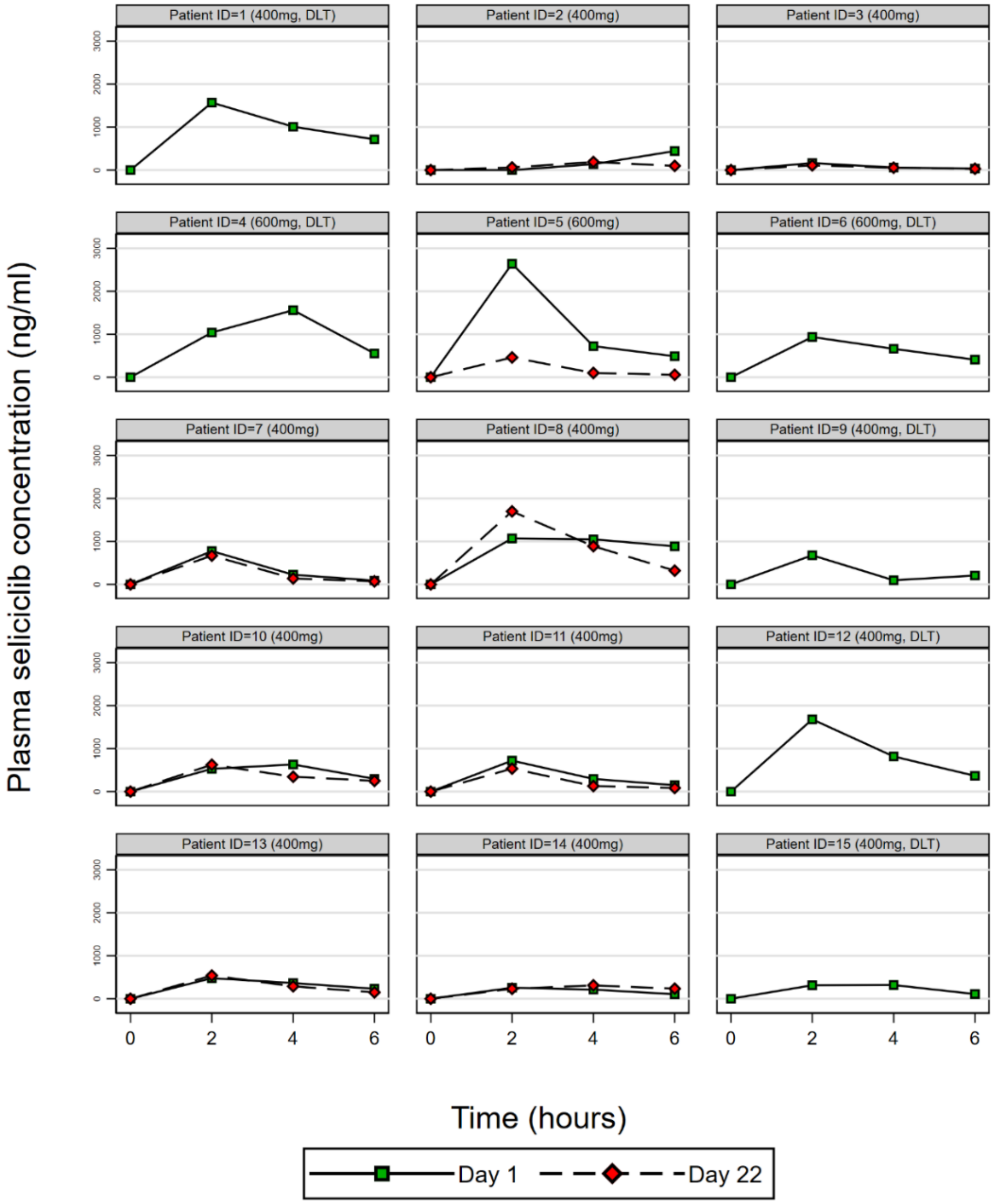

Figure S2. *Plasma seliciclib concentrations 0, 2, 4 and 6 hours after dosing on days 1 (green; unbroken line) and 22 (red; broken line, unavailable for patients who experienced DLTs). Headers show prescribed daily dose and indicate if DLT occurred. Cohorts 1-5 are depicted by row (uppermost = cohort 1).*

Figure S3

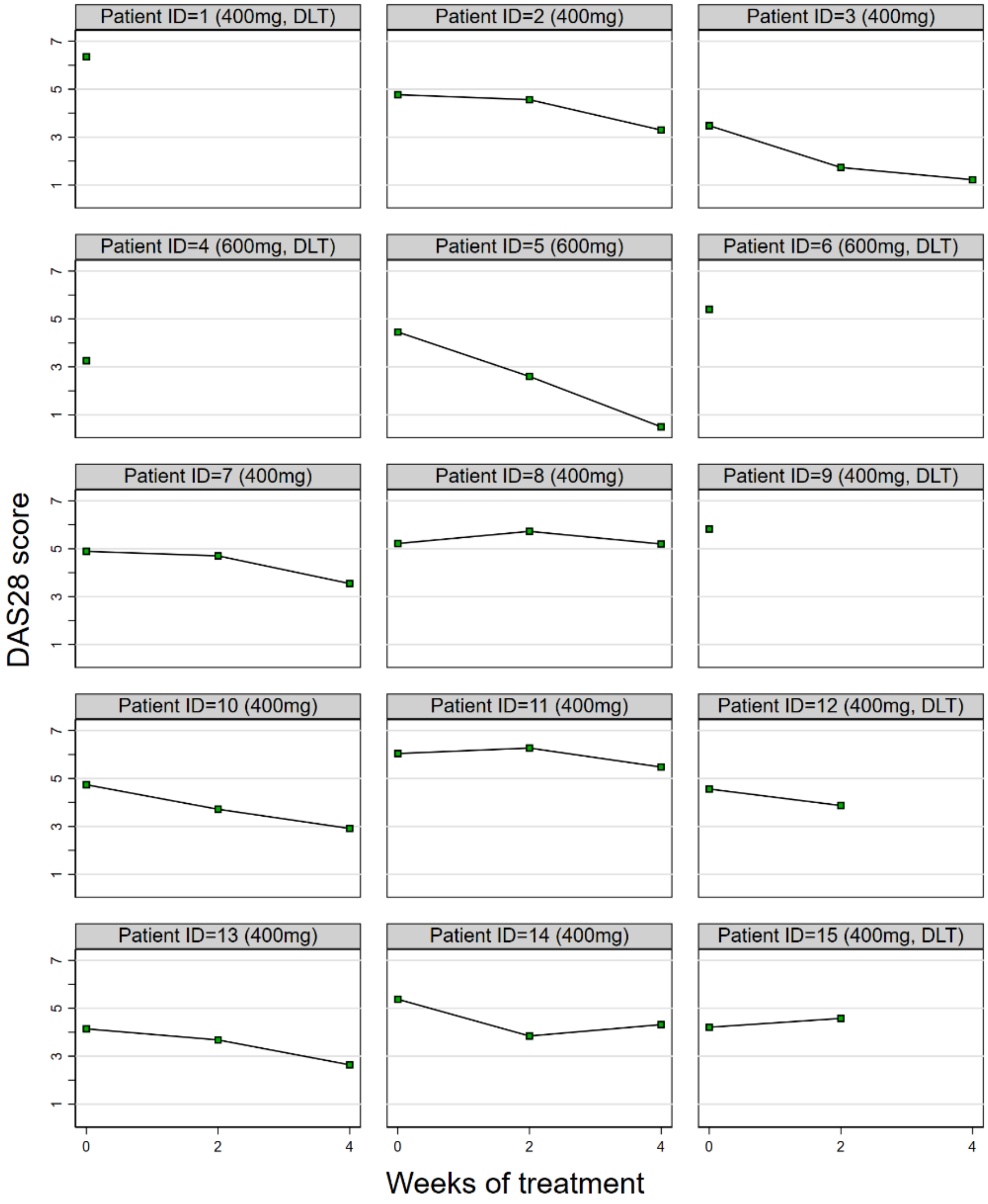

***Supplementary Figure S3**. DAS28-CRP scores obtained prior to seliciclib treatment and at indicated time points following treatment initiation for all trial participants where data available. Headers show prescribed daily dose and indicate if DLT occurred. Cohorts 1-5 are depicted by row (uppermost = cohort 1).*

Figure S4

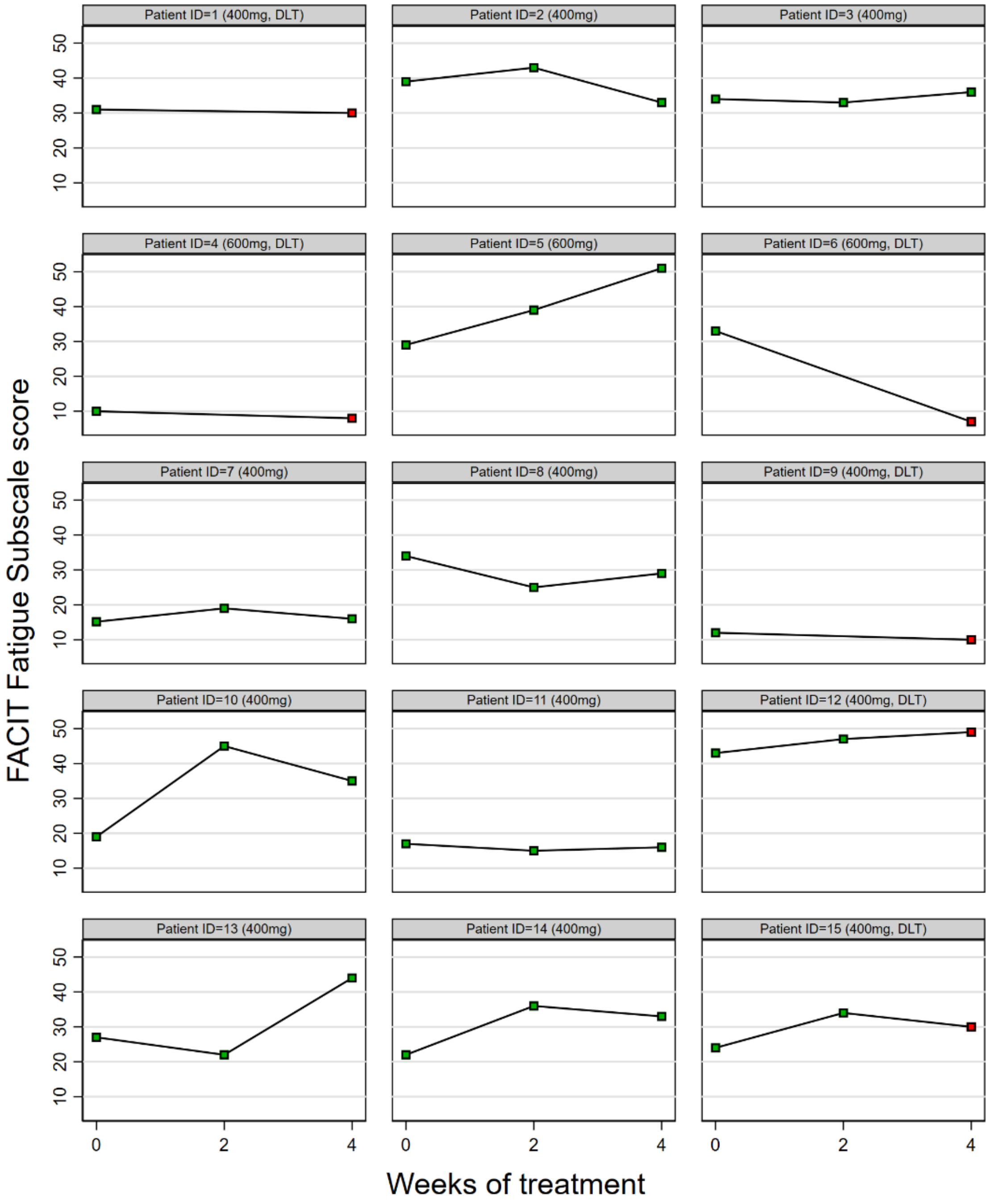

Supplementary Figure S4*. FACIT Fatigue subscale score by patient at the indicated time points following treatment initiation where data available; Higher scores indicate lower reported fatigue. Data points at 4 weeks are depicted in either green (measured after 4 weeks' completed therapy) or red (measured at visit following treatment cessation after DLT). Headers show prescribed daily dose and indicate if DLT occurred. Cohorts 1-5 are depicted by row (uppermost = cohort 1).*
